# Genetic risk scores: are they important for diabetes management? results from multiple cross-sectional studies

**DOI:** 10.1186/s13098-023-01204-9

**Published:** 2023-11-10

**Authors:** Sureka Nagarajah, Abdullah Alkandari, Pedro Marques-Vidal

**Affiliations:** 1 Department of Medicine, Internal Medicine, Lausanne University Hospital and University of Lausanne, Office BH10-642, 46 Rue du Bugnon, 1011 Lausanne, Switzerland; 2 Dasman Diabetes Institute, Kuwait City, Kuwait

**Keywords:** Type 2 diabetes, Genetic risk scores, Treatment, Control, Epidemiology

## Abstract

**Background:**

Several genetic risk scores (GRS) for type 2 diabetes (T2DM) have been published, but not replicated. We aimed to 1) replicate previous findings on the association between GRS on prevalence of T2DM and 2) assess the association between GRS and T2DM management in a sample of community-dwelling people from Switzerland.

**Methods:**

Four waves from a prospective study conducted in Lausanne. Seven GRS related to T2DM were selected, and compared between participants with and without T2DM, and between controlled and uncontrolled participants treated for T2DM.

**Results:**

Data from 5426, 4017, 2873 and 2170 participants from the baseline, first, second and third follow-ups, respectively, was used. In all study periods, participants with T2DM scored higher than participants without T2DM in six out of seven GRS. Data from 367, 437, 285 and 207 participants with T2DM was used. In all study periods, approximately half of participants treated for T2DM did not achieve adequate fasting blood glucose or HbA_1_c levels, and no difference between controlled and uncontrolled participants was found for all seven GRS. Power analyses showed that most GRS needed a sample size above 1000 to consider the difference between controlled and uncontrolled participants as statistically significant at p = 0.05.

**Conclusion:**

In this study, we confirmed the association between most published GRS and diabetes. Conversely, no consistent association between GRS and diabetes control was found. Use of GRS to manage patients with T2DM in clinical practice is not justified.

**Supplementary Information:**

The online version contains supplementary material available at 10.1186/s13098-023-01204-9.

## Background

Prevalence of diabetes mellitus type 2 (T2DM) has more than doubled globally [1, 2]. Guidelines regarding the management of T2DM have been issued [3, 4], but still half of treated subjects with diabetes remain inadequately controlled [5, 6].

Recently, several studies have identified a range of genetic markers associated with T2DM or its management [7, 8]. Several studies suggested that some polymorphisms might be related to response to antidiabetic drugs [9–11], although other studies found no significant association [12]. These discrepancies between the different authors may be because the genetic risk scores (GRS) used are different. For example, the GRS proposed by Andersson et al. needs a weighting of single nucleotide polymorphisms (SNPs) [7], unlike Martono et al.’s score, which has no weighting [12]. The number of SNPs in the GRS also vary considerably: Pechlivanis et al. propose a GRS with 100 SNPs [13], while Wang et al. propose a GRS with only 17 SNPs [14]. Pechlivanis et al. reported no association between genetic markers and T2DM, while Wang et al. reported that the GRS had an impact on diabetes management [13, 14]. Interestingly, the 16-SNP GRS of Wang et al. included nine SNPs that were part of the GRS by Pechlivanis et al. There are also differences between the authors regarding the number of participants in their study. Andersson et al. worked on a sample size of 5850 participants; Wang et al. and Pechlivanis et al. had a sample size of 5712 and 4814 participants, respectively [7, 13]. Conversely, Liu et al., Szczerbinski et al. and Martono et al. had sample sizes of 1385, 1195 and 696 participants, respectively [12, 15–17]. Finally, all authors did not replicate their findings in other cohorts. Andersson et al. concluded that the results could not necessarily be transferred to other populations [7], while Liu et al. reported that more external studies are necessary to validate his results and generalize on other populations [16].

Hence, we aimed to replicate the results of the previous studies by assessing the association between different GRS and prevalence of T2DM. We also assessed the association between the different GRS and control of T2DM among treated participants in a sample of community-dwelling people from Switzerland. Our hypotheses were that 1) participants with T2DM would present higher levels for all types of GRS than participants without T2DM, and 2) participants treated for T2DM but uncontrolled regarding their disease would also present higher levels for all types of GRS than participants treated but controlled.

## Methods

### Participants

The CoLaus|PsyCoLaus study is a population-based study investigating the epidemiology and genetic determinants of psychiatric and cardiovascular disease in Lausanne, Switzerland [18]. Briefly, a representative sample was collected through a simple, non-stratified random sampling of 19,830 individuals (35% of the source population) aged between 35 and 75. The baseline study was conducted between June 2003 and May 2006; the first follow-up was performed between April 2009 and September 2012; the second follow-up was performed between May 2014 and April 2017 and the third follow-up was performed between April 2018 and May 2021.

Within each survey, participants answered questionnaires regarding their lifestyle, socio-economic status, medications, and diseases. Several anthropometric measurements were performed, and fasting blood samples were obtained.

### Genotyping

Genome-wide genotyping was performed using the Affymetrix 500 K SNP array. Nuclear DNA was extracted from the whole blood of all participants. Genotypes were called using BRLMM. Duplicate individuals, and first and second-degree relatives, were identified and the removed by computing estimates pairwise genomic kinship coefficients, using KING [19].

Subjects were excluded from the analysis in case of inconsistency between self-reported sex and genetic data, a genotype call rate < 90%, or inconsistencies of genotyping results in duplicate samples. Quality control for SNPs was performed using the following criteria: monomorphic (or with minor allele frequency (MAF) < 1%), call rates < 90%, deviation from the Hardy–Weinberg equilibrium (HWE) (p < 1 × 10^-6^). Phased haplotypes were generated using SHAPEIT2 [20, 21]. Imputation was performed using minimac3 [22] and the Haplotype Reference Consortium (HRC version r1.1) hosted on the Michigan Imputation Server [23].

### Genetic risk scores

We conducted a literature search regarding genetic risk scores (GRS) for T2DM. Seven GRS related to T2DM were selected; their characteristics are summarized in supplementary tables 1 to 7. Analysis was conducted as follows: firstly, we listed all genes and SNPs on an Excel table; secondly, we pooled genes and SNPs that repeated multiple times on a single line. When the same SNP could be attributed to two different genes, we selected the one with the highest relevance score according to the GeneCards database (www.genecards.org, accessed August 22^nd^, 2022). We also searched for SNPs that were common to at least five out of seven of the analysed GRS, because there were only two genes that were common to the seven scores (supplementary tables 8 and 9). This led to 12 out of 123 SNPs, which were used to create a “short” score. A “large” score was also built considering all 123 SNPs. Both the “short” and the “large” scores were unweighted.

### Diabetes assessment

Glucose levels were assessed at all survey periods by glucose hexokinase (maximum inter and intra-batch CV: 1.6 and 0.8%) and glycated haemoglobin levels were measured at the second and third follow-ups by high performance liquid chromatography using Bio-Rad, D-10TM system, with measurement range 3.8% (18 mmol/mol) to 18.5% (179 mmol/mol).

Diabetes status was defined as a presence of antidiabetic treatment or a fasting blood glucose ≥ 7 mmol/L (definition 1) or a glycated haemoglobin level ≥ 48 mmol/mol (6.5%, definition 2). Controlled diabetes was defined among participants treated with antidiabetic drugs if a fasting plasma glucose was < 7 mmol/L (definition 1) or a glycated haemoglobin level < 48 mmol/mol (6.5%, definition 2).

### Covariates

Smoking was self-reported and categorized into never, former, and current. Educational level was categorized into high (university), middle (high school) and low (mandatory + apprenticeship). Marital status and alcohol consumption were defined as binary variables (yes/no).

Body weight and height were measured with participants barefoot and in light indoor clothes. Body weight was measured in kilograms to the nearest 100 g using a Seca^®^ scale (Hamburg, Germany). Height was measured to the nearest 5 mm using a Seca^®^ (Hamburg, Germany) height gauge. Body mass index (BMI) was computed and categorized into normal (BMI < 25 kg/m^2^), overweight (BMI ≥ 25 and < 30 kg/m^2^) and obese (≥ 30 kg/m^2^).

### Inclusion and exclusion criteria

Participants were included if they had genetic data. Participants with missing data for the main variables or covariates (glucose, smoking, BMI, education, marital status…) were excluded.

### Statistical analysis

Statistical analyses were conducted using Stata version 16.1 (Stata Corp, College Station, TX, USA). Results were expressed as number of participants (percentage) for categorical variables and as average ± standard deviation for continuous variables. Analyses were performed as follows: first, the GRS were compared between participants with and without T2DM to ascertain that the GRS were associated with T2DM. Second, the GRS were compared between participants treated for diabetes according to their condition (controlled or not). Bivariate analyses were conducted using student’s t-test, and multivariable analyses were conducted using analysis of covariance (ANCOVA) adjusting for age (continuous), gender, marital status (yes, no), educational level (high, medium, low), smoking categories (never, former, current), alcohol consumption (yes, no) and BMI categories (normal, overweight, obese). Results of the multivariable analyses were presented as adjusted mean ± standard error. Statistical significance was considered for a two-sided test with p < 0.05.

The main analyses were performed using plasma glucose as diagnosis and control marker (definition 1). A second set of analyses was conducted using glycated haemoglobin (definition 2). Power analyses were conducted by computing the total sample size needed to detect the difference between controlled and uncontrolled participants, using a p-value of 0.05, a power of 0.80, a ratio uncontrolled to controlled participants of 2, and the mean standard deviation between controlled and uncontrolled participants.

## Result

### Characteristics of participants

The selection procedure for each study period is summarized in supplementary Fig. 1 and the characteristics of included and excluded participants according to survey period are summarized in supplementary table 10. Excluded participants were younger, with a higher education level, less frequently alcohol consumers, and more frequently current smokers than included participants. No consistent differences were found between excluded and included participants regarding gender, marital status, or BMI.

### Genetic risk scores for diabetes

The values of the different GRS according to presence or absence of diabetes as defined by fasting glucose and for each survey period are summarized in supplementary table 11. Participants with diabetes had significantly higher scores than participants without diabetes in six out of seven GRS. Participants with diabetes also scored higher for the short GRS but not for the large score. All GRS analysed were higher in participants with diabetes compared to participants without diabetes, although the distributions tended to overlap considerably (supplementary Fig. 2).

The values of the different GRS according to presence or absence of diabetes as defined by glycated haemoglobin and for each survey period are summarized in supplementary table 12. Participants with diabetes had significantly higher scores than participants without diabetes in four out of seven established GRS. Participants with diabetes also scored higher for the short GRS but not for the large score. However, all GRS analysed were higher in participants with T2DM compared to participants without T2DM.

### Effect of genetic risk scores for diabetes according to diabetes treatment and control

The values of the different GRS among participants treated for diabetes according to diabetes control according to glucose levels (definition 1) and for each study period are summarized in Table 1. For all study periods and GRS, no significant difference was found between controlled and uncontrolled groups. Similar findings were obtained in the multivariable analyses (Table 1).

**Table 1:** comparison of diabetes genetic risk scores according to diabetes control as defined by glucose levels, by survey period, CoLaus study, Lausanne, Switzerland

		Bivariate			Multivariable	
Period/Score	Not controlled	Controlled	P-value	Not controlled	Controlled	P-value
Baseline (N)	150	79		150	79	
Andersson	41.2 ± 5.2	40.7 ± 4.7	0.499	41.2 ± 0.4	40.6 ± 0.6	0.356
Martono	62.1 ± 4.5	61.6 ± 4.7	0.377	62.1 ± 0.4	61.6 ± 0.5	0.447
Szcerbinski	0.53 ± 0.44	0.53 ± 0.41	0.944	0.52 ± 0.04	0.54 ± 0.05	0.833
Werissa	17.5 ± 3.4	17.7 ± 3.3	0.555	17.5 ± 0.3	17.8 ± 0.4	0.534
Liu	2.86 ± 0.32	2.91 ± 0.30	0.274	2.86 ± 0.03	2.91 ± 0.04	0.251
Pechlivanis	108 ± 6	109 ± 6	0.825	108 ± 1	108 ± 1	0.938
Wang	1.52 ± 0.38	1.55 ± 0.38	0.571	1.52 ± 0.03	1.55 ± 0.04	0.632
Large score	130 ± 10	128 ± 10	0.327	130 ± 1	128 ± 1	0.339
Short score	10.1 ± 2.4	10.0 ± 2.4	0.810	10.1 ± 0.2	9.9 ± 0.3	0.574
First follow-up (N)	153	83		153	83	
Andersson	40.1 ± 4.7	40.7 ± 5.3	0.362	40.2 ± 0.4	40.5 ± 0.5	0.633
Martono	62.6 ± 4.2	61.5 ± 4.6	0.082	62.6 ± 0.3	61.5 ± 0.5	0.087
Szcerbinski	0.56 ± 0.41	0.49 ± 0.40	0.221	0.56 ± 0.03	0.49 ± 0.04	0.233
Werissa	17.5 ± 3.3	17.1 ± 2.9	0.356	17.6 ± 0.3	17.1 ± 0.3	0.309
Liu	2.89 ± 0.33	2.85 ± 0.35	0.331	2.89 ± 0.03	2.85 ± 0.04	0.411
Pechlivanis	109 ± 6	107 ± 7	0.093	108 ± 1	107 ± 1	0.109
Wang	1.54 ± 0.39	1.54 ± 0.38	0.877	1.54 ± 0.03	1.54 ± 0.04	0.946
Large score	127 ± 10	128 ± 11	0.392	127 ± 1	128 ± 1	0.768
Short score	9.8 ± 2.3	9.9 ± 2.4	0.873	9.8 ± 0.2	9.8 ± 0.3	0.833
Second follow-up (N)	110	114		110	114	
Andersson	40.3 ± 4.6	40.2 ± 5.0	0.933	40.3 ± 0.5	40.2 ± 0.5	0.913
Martono	61.9 ± 4.7	61.4 ± 4.5	0.382	62.0 ± 0.5	61.5 ± 0.5	0.492
Szcerbinski	0.54 ± 0.40	0.49 ± 0.39	0.338	0.56 ± 0.04	0.49 ± 0.04	0.207
Werissa	17.6 ± 3.4	17.4 ± 3.0	0.678	17.8 ± 0.3	17.4 ± 0.3	0.447
Liu	2.88 ± 0.36	2.82 ± 0.30	0.209	2.89 ± 0.03	2.82 ± 0.03	0.166
Pechlivanis	109 ± 6	107 ± 6	0.034	109 ± 1	108 ± 1	0.064
Wang	1.49 ± 0.40	1.47 ± 0.37	0.720	1.51 ± 0.04	1.47 ± 0.04	0.473
Large score	127 ± 10	127 ± 11	0.494	126 ± 1	127 ± 1	0.420
Short score	9.8 ± 2.2	9.8 ± 2.4	0.866	9.8 ± 0.2	9.7 ± 0.2	0.906
Third follow-up (N)	92	65		92	65	
Andersson	39.7 ± 5.2	40.2 ± 4.0	0.518	39.9 ± 0.5	40.0 ± 0.6	0.857
Martono	61.8 ± 4.9	62.2 ± 5.0	0.578	61.8 ± 0.5	62.2 ± 0.6	0.586
Szcerbinski	0.53 ± 0.46	0.50 ± 0.39	0.586	0.55 ± 0.04	0.48 ± 0.05	0.320
Werissa	17.3 ± 3.7	17.6 ± 3.1	0.559	17.3 ± 0.4	17.5 ± 0.4	0.714
Liu	2.85 ± 0.39	2.83 ± 0.31	0.622	2.87 ± 0.04	2.81 ± 0.04	0.339
Pechlivanis	108 ± 6	110 ± 7	0.094	108 ± 1	110 ± 1	0.079
Wang	1.51 ± 0.41	1.45 ± 0.36	0.328	1.52 ± 0.04	1.44 ± 0.05	0.255
Large score	126 ± 10	126 ± 9	0.742	126 ± 1	126 ± 1	0.892
Short score	9.8 ± 2.4	9.5 ± 2.1	0.519	9.8 ± 0.2	9.5 ± 0.3	0.352

Results are expressed as average ± standard deviation for the bivariate analyses and as multivariate adjusted mean ± standard error for multivariate analyses. Between-group comparisons performed using student t-test for bivariate analyses and ANCOVA adjusting for age (continuous), gender, marital status (yes, no), educational level (high, medium, low), smoking categories (never, former, current), alcohol consumption (yes, no) and body mass index categories (normal, overweight, obese)

The values of the different GRS among participants treated for diabetes according to diabetes control according to HbA_1_c levels (definition 2) and for each study period are summarized in Table 2. Except for the higher value of the Andersson’s GRS among controlled participants in the third follow-up, no significant difference was found between controlled and uncontrolled groups for all other GRS in both survey periods. Similar findings were obtained in the multivariable analyses (Table 2).

**Table 2:** comparison of diabetes genetic risk scores according to diabetes control as defined by HbA_1_c levels, by survey period, CoLaus study, Lausanne, Switzerland

		Bivariate			Multivariable	
Period/Score	Not controlled	Controlled	P-value	Not controlled	Controlled	P-value
Second follow-up (N)	132	92		132	92	
Andersson	40.0 ± 4.6	40.7 ± 5.1	0.291	40.0 ± 0.4	40.6 ± 0.5	0.423
Martono	62.1 ± 4.7	61.0 ± 4.5	0.085	62.2 ± 0.4	61.1 ± 0.5	0.105
Szcerbinski	0.53 ± 0.41	0.50 ± 0.37	0.585	0.54 ± 0.04	0.50 ± 0.05	0.524
Werissa	17.5 ± 3.3	17.5 ± 3.1	0.992	17.6 ± 0.3	17.5 ± 0.4	0.759
Liu	2.88 ± 0.34	2.80 ± 0.32	0.100	2.89 ± 0.03	2.80 ± 0.04	0.067
Pechlivanis	109 ± 6	107 ± 6	0.069	109 ± 1	107 ± 1	0.129
Wang	1.49 ± 0.38	1.47 ± 0.38	0.748	1.50 ± 0.03	1.47 ± 0.04	0.542
Large score	126 ± 9	128 ± 11	0.134	126 ± 1	128 ± 1	0.070
Short score	9.8 ± 2.2	9.8 ± 2.4	0.986	9.8 ± 0.2	9.7 ± 0.3	0.840
Third follow-up (N)	95	62		95	62	
Andersson	39.2 ± 4.5	41.1 ± 4.8	0.011	39.2 ± 0.5	41.0 ± 0.6	0.023
Martono	61.8 ± 4.8	62.2 ± 5.3	0.579	61.7 ± 0.5	62.3 ± 0.6	0.509
Szcerbinski	0.48 ± 0.43	0.58 ± 0.42	0.154	0.48 ± 0.04	0.58 ± 0.05	0.149
Werissa	17.1 ± 3.6	17.9 ± 3.1	0.189	17.1 ± 0.3	17.9 ± 0.4	0.188
Liu	2.83 ± 0.37	2.86 ± 0.33	0.669	2.83 ± 0.04	2.86 ± 0.05	0.671
Pechlivanis	109 ± 6	109 ± 7	0.880	109 ± 1	109 ± 1	0.582
Wang	1.52 ± 0.42	1.43 ± 0.34	0.175	1.52 ± 0.04	1.43 ± 0.05	0.172
Large score	125 ± 10	127 ± 10	0.250	125 ± 1	127 ± 1	0.351
Short score	9.4 ± 2.2	10.0 ± 2.4	0.114	9.4 ± 0.2	10.0 ± 0.3	0.123

Results are expressed as average ± standard deviation for the bivariate analyses and as multivariate adjusted mean ± standard error for multivariate analyses. Between-group comparisons performed using student t-test for bivariate analyses and ANCOVA adjusting for age (continuous), gender, marital status (yes, no), educational level (high, medium, low), smoking categories (never, former, current), alcohol consumption (yes, no) and body mass index categories (normal, overweight, obese)

The results of the power analyses are summarized in supplementary table 13. Except for Pechlivanis et al.’s score, most GRS needed a sample size above 1000 to consider the difference between controlled and uncontrolled participants as statistically significant at p = 0.05.

## Discussion

In this study, we confirmed the association between most GRS and diabetes. Conversely, no consistent association between GRS and diabetes control was found.

### Genetic risk scores for diabetes

Participants with T2DM as defined by fasting plasma glucose scored higher in six out of seven published GRS and for the short GRS. Our results replicate those of the previous studies [12–17], with the exception of the study by Andersson et al. [7], the results of which we failed to replicate. A possible explanation is that Andersson et al. [7] sought to see the impact of changes in lifestyle and BMI on the glycaemic response via GRS. Therefore, the genes involved may have an impact only with the modification of lifestyle and BMI. Interestingly, the small GRS composed of the most common SNPs also produced significant results, suggesting that a small number of SNPs might suffice to create a GRS associated with diabetes.

Noteworthy, and albeit being statistically significant, most differences in GRS between participants with and without diabetes were small and the distributions overlapped, suggesting that the GRS are not a good tool for the screening or diagnosis of diabetes.

### Effect of genetic risk scores for diabetes according to diabetes treatment and control

No differences were found between treated participants according to diabetes control. Our results are in agreement with the only study that assessed the associations between GRS and diabetes control, although for a HbA1c < 7% (53 mmol/mol) [12]. Further, the power analysis showed that it is necessary to calculate the GRS in at least 1000 patients with diabetes to obtain a significant difference between controlled and uncontrolled participants. The exception was the GRS by Pechlivanis et al.'s [13], where the number needed to screen was smaller than for the other GRS. However, in clinical practice, no general practitioner or even endocrinologist-diabetologist will have a patient population of this size. Therefore, the use of GRS to assess response to treatment as well as to identify patients at risk of having inadequate control of their diabetes is not justified. Rather than genotyping and computing GRS in patients with T2DM, practitioners should focus on patient compliance or on adapting treatment, as in this study over 50% of patients treated for diabetes was not adequately controlled.

Several gene*environment interactions have been suggested in the development of T2DM, namely regarding pollutants such as arsenic [24] or polychlorinated biphenyls [25]. Other interactions with lifestyle such as physical activity [26, 27], smoking [27, 28] and diet [27] have been reported, although most failed to be replicated [29–32]. Hence, it would be important that further studies analyse the effect of gene*environment interactions on the incidence and management of T2DM.

### Strengths and limitations

This study was the first to assess the association between a series of GRS and prevalence of diabetes. It thus confirms the results of previous studies in a different setting. It is also one of the very few studies assessing the association between GRS and control of diabetes.

This study also has some limitations. First, it was conducted in a single location, and results might not be generalizable to other settings. Still, they provide valuable information for clinicians in Switzerland. Second, the GRS might not be fully adapted to the Swiss population due to genetic variations; still, we confirmed the association with almost all GRS and diabetes, and our sample has a relatively wide genetic variability [33]. Finally, the sample size might have been too small to detect differences between controlled and uncontrolled participants; still, the power analyses showed that, for most GRS, very large sample sizes would be needed to achieve significance.

To conclude, we confirmed the association between most GRS and diabetes. Conversely, no consistent association between GRS and diabetes control was found. Use of GRS to manage patients with T2DM in clinical practice is not justified.

### Supplementary Information

**Additional file 1: Figure S1.** flowchart of participant’s selection. GRS, genetic risk score; FU, follow-up. Study periods: baseline, June 2003 to May 2006; first follow-up, April 2009 to September 2012; second follow-up, May 2014 to April 2017; third follow-up, April 2018 to May 2021.

**Additional file 2: Figure S2.** distribution of the genetic risk scores according to presence or absence of diabetes. Panel **A** Andersson score; **B** Liu score; **C** Martono score; **D** Pechlivanis score; **E** Szczerbinski score; **F** Wang score; **G** Werissa score; **H** Full score; **I** Short score.

**Additional file 3: Table S1.** Components of the genetic risk score for T2DM as proposed by Andersson et al., 2013. **Table S2.** Components of the genetic risk score for T2DM as proposed by Martono et al., 2019. **Table S3.** Components of the genetic risk score for T2DM as proposed by Szczerbinski et al., 2019. **Table S4.** Components of the genetic risk score for T2DM as proposed by Werissa et al., 2019. **Table S5.** Components of the genetic risk score for T2DM as proposed by Liu et al., 2021. **Table S6.** Components of the genetic risk score for T2DM as proposed by Pechlivanis et al., 2021. **Table S7.** Components of the genetic risk score for T2DM as proposed by Wang et al., 2021. **Table S8.** distribution of genes and SNPs within the different genetic risk scores for T2DM. **Table S9.** distribution of genes within the different genetic risk scores for T2DM. **Table S10.** comparison between included and excluded participants, by survey period, CoLaus study, Lausanne, Switzerland. **Table S11.** diabetes risk scores according to diabetes status defined by glucose levels, by survey period, CoLaus study, Lausanne, Switzerland. **Table S12**. diabetes risk scores according to diabetes status defined by HbA1c levels, by survey period, CoLaus study, Lausanne, Switzerland. **Table S13.** sample sizes needed to consider the difference between controlled and not-controlled groups as significant at p < 0.05.

## Data Availability

The data of CoLaus|PsyCoLaus study used in this article cannot be fully shared as they contain potentially sensitive personal information on participants. According to the Ethics Committee for Research of the Canton of Vaud, sharing these data would be a violation of the Swiss legislation with respect to privacy protection. However, coded individual-level data that do not allow researchers to identify participants are available upon request to researchers who meet the criteria for data sharing of the CoLaus|PsyCoLaus Datacenter (CHUV, Lausanne, Switzerland). Any researcher affiliated to a public or private research institution who complies with the CoLaus|PsyCoLaus standards can submit a research application to research.colaus@chuv.ch or research.psycolaus@chuv.ch. Proposals requiring baseline data only, will be evaluated by the baseline (local) Scientific Committee (SC) of the CoLaus and PsyCoLaus studies. Proposals requiring follow-up data will be evaluated by the follow-up (multicentric) SC of the CoLaus|PsyCoLaus cohort study. Detailed instructions for gaining access to the CoLaus|PsyCoLaus data used in this study are available at www.colaus-psycolaus.ch/professionals/how-to-collaborate/.

## Abbreviations

ANOVA: Analysis of variance
BMI: Body mass index
GRS: Genetic risk score
SNP: Single nucleotide polymorphism
T2DM: Type 2 diabetes mellitus
