# Supplementary material for: Genetic risk scores: are they important for diabetes management? results from multiple cross-sectional studies
Source: Diabetol Metab Syndr. 2023 Nov 10;15:227. doi: 10.1186/s13098-023-01204-9 (PMC10636836; doi:10.1186/s13098-023-01204-9)
Supplement: Supplementary file 1 — Additional file 1: Figure S1. flowchart of participant’s selection. GRS, genetic risk score; FU, follow-up. Study periods: baseline, June 2003 to May 2006; first follow-up, April 2009 to September 2012; second follow-up, May 2014 to April 2017; third follow-up, April 2018 to May 2021. [file 13098_2023_1204_MOESM1_ESM.pptx]

## Slide 1
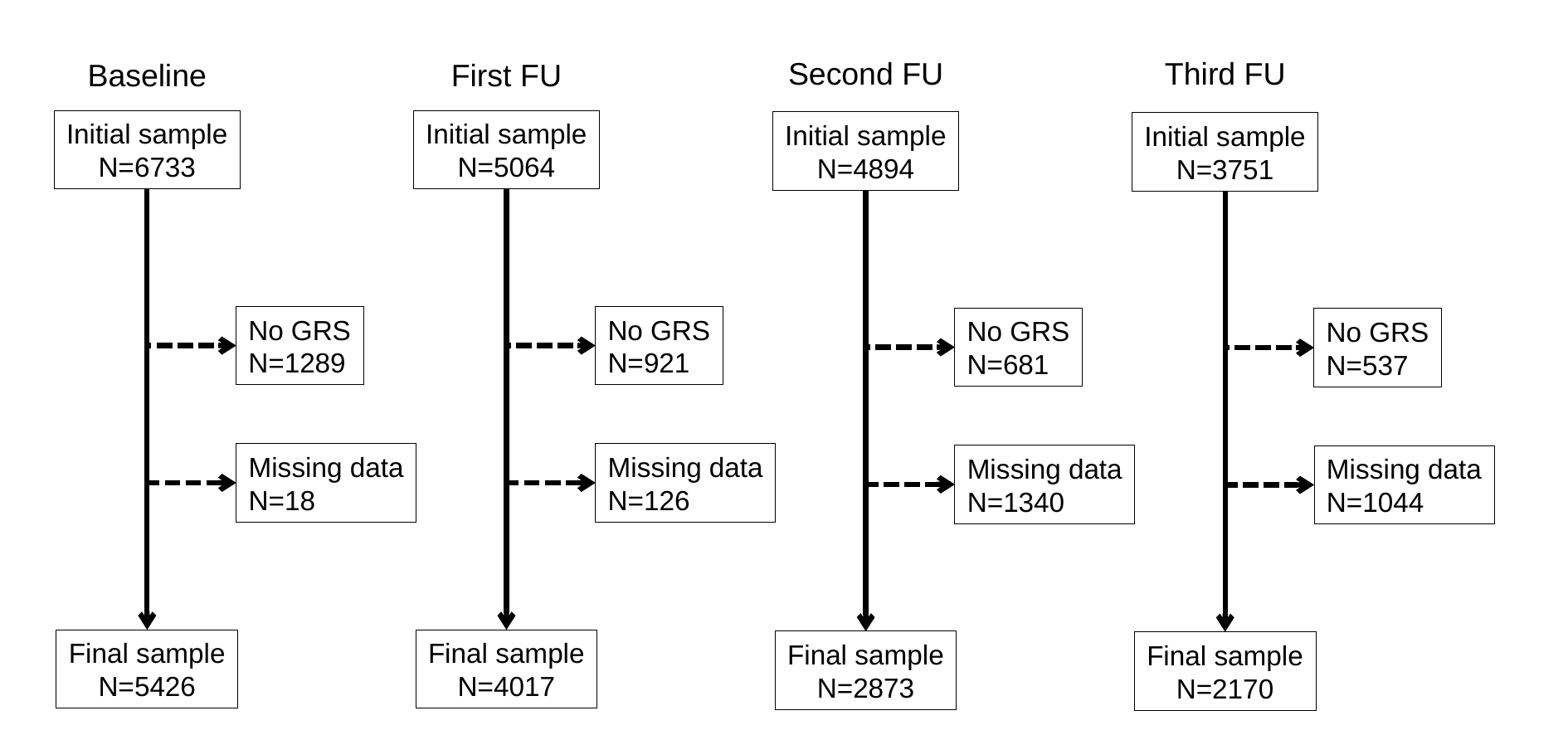

Third FU
Initial sample
N=3751
No GRS
N=537
Missing data
N=1044
Final sample
N=2170
Second FU
Initial sample
N=4894
No GRS
N=681
Missing data
N=1340
Final sample
N=2873
Baseline
Initial sample
N=6733
No GRS
N=1289
Missing data
N=18
Final sample
N=5426
First FU
Initial sample
N=5064
No GRS
N=921
Missing data
N=126
Final sample
N=4017
