# Supplementary figures and images for: Genetic risk scores: are they important for diabetes management? results from multiple cross-sectional studies
Source: Diabetol Metab Syndr. 2023 Nov 10;15:227. doi: 10.1186/s13098-023-01204-9 (PMC10636836; doi:10.1186/s13098-023-01204-9)

## Slide 1
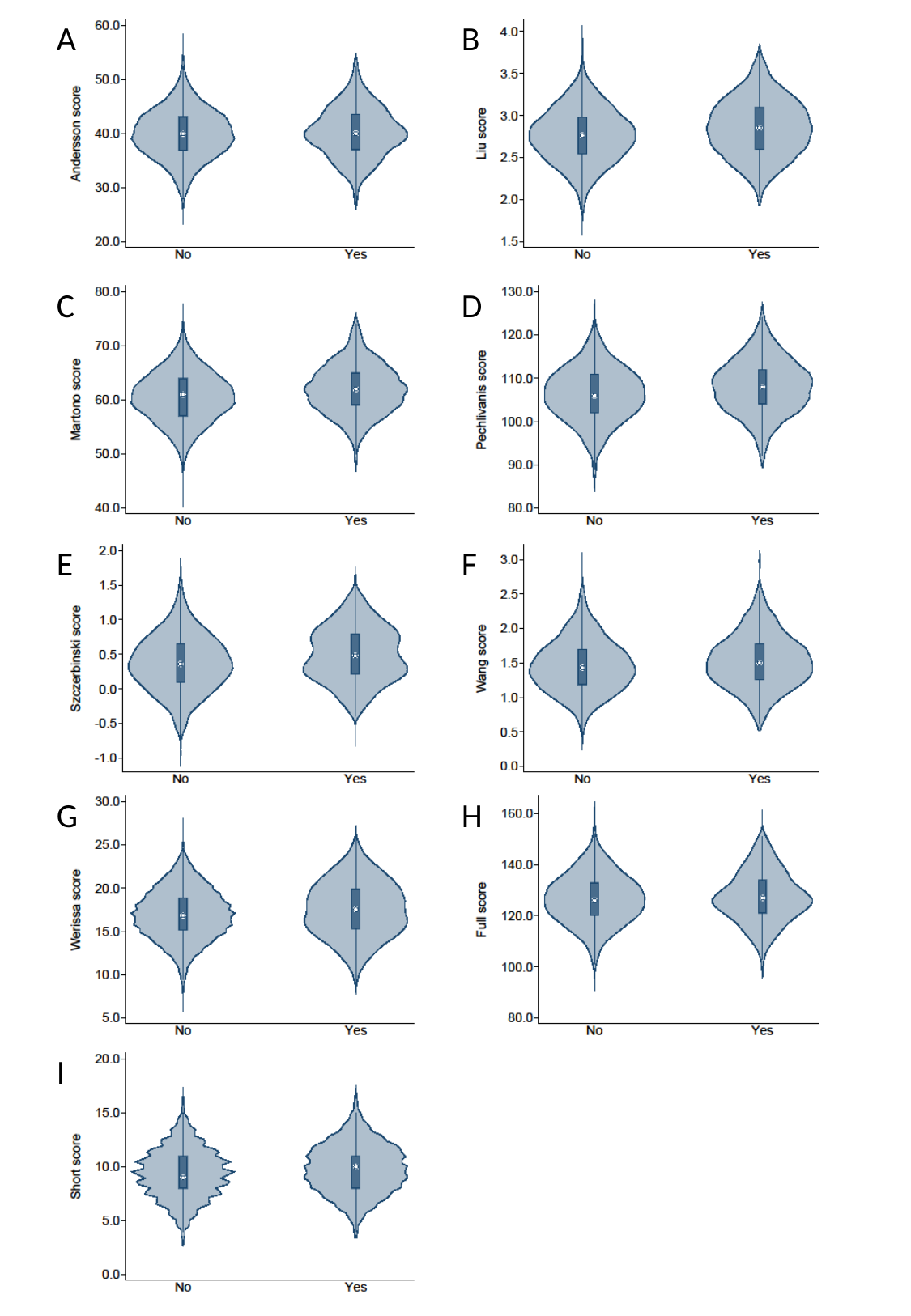

A
B
C
D
E
F
G
H
I

Supplement: Supplementary file 2 — Additional file 2: Figure S2. distribution of the genetic risk scores according to presence or absence of diabetes. Panel A Andersson score; B Liu score; C Martono score; D Pechlivanis score; E Szczerbinski score; F Wang score; G Werissa score; H Full score; I Short score. [file 13098_2023_1204_MOESM2_ESM.pptx]
